# Systematic Analysis and Functional Validation of Citrus XTH Genes Reveal the Role of Csxth04 in Citrus Bacterial Canker Resistance and Tolerance

**DOI:** 10.3389/fpls.2019.01109

**Published:** 2019-09-27

**Authors:** Qiang Li, Anhua Hu, Wanfu Dou, Jingjing Qi, Qin Long, Xiuping Zou, Tiangang Lei, Lixiao Yao, Yongrui He, Shanchun Chen

**Affiliations:** Citrus Research Institute, Southwest University, Chinese Academy of Agricultural Sciences, Chongqing, China

**Keywords:** xyloglucan endotransglucosylase/hydrolases (XTHs), sweet orange, Citrus sinensis, citrus bacterial canker (CBC), *Xanthomonas citri* subsp. *citri* (Xcc)

## Abstract

In this study, we performed a comprehensive survey of xyloglucan endotransglucosylase/hydrolase (XTH) and a functional validation of *Citrus sinensis* (Cs) XTH genes to provide new insights into the involvement of XTHs in *Xanthomonas citri* subsp. *citri* (Xcc) infection. From the genome of sweet orange, 34 CsXTH genes with XTH characteristic domains were identified and clustered into groups I/II, IIIA, and IIIB. Except for chromosome 9, the CsXTH genes were unevenly distributed and duplicated among all chromosomes, identifying a CsXTH duplication hot spot on chromosome 4. With Xcc induction, a group of citrus canker-related CsXTHs were detected. CsXTH04 was identified as a putative candidate gene, which is up-regulated in citrus bacterial canker (CBC)-resistant varieties and induced by exogenous treatment with salicylic acid (SA) and methyl jasmonate (MeJA). CsXTH04 overexpression conferred CBC susceptibility to transgenic citrus, while CsXTH04 silencing conferred CBC resistance. Taken together, the annotation of the CsXTH family provides an initial basis for the functional and evolutionary study of this family as potential CBC-susceptible genes. CsXTH04, validated in this study, can be used in citrus breeding to improve CBC resistance.

## Introduction

The cell wall is a dynamic architecture of celluloses, pectic polysaccharides, enzymes, and many structural proteins (Yokoyama and Nishitani, 2001). Under biotic and abiotic stresses (e.g., bacterial pathogens and salinity), the cell wall undergoes physiological and molecular mechanical changes along with cellular morphology variations, changes in metabolism, and non-protein component variations (Cosgrove, 1997). Xyloglucans cross-link the adjacent cellulose microfibrils with hydrogen bonds, which confers strength to the plant cell wall (Rose et al., 2002). An array of enzymes regulate cell wall processes, including expansins (EXPs) (Li et al., 2003), endo-1,4-*b*-d-endoglucanase (EGases) (del Campillo, 1999; del Campillo et al., 2004), and xyloglucan endotransglucosylase/hydrolases (XTHs) (Rose et al., 2002; Maldonado-Mendoza et al., 2005; Xuan et al., 2016). These enzymes modulate the shape of the cell wall with primary or secondary wall-loosening mechanisms (Li et al., 2003). In the processes of cell wall construction, degradation, and extension, xyloglucan is cleaved and reforms xyloglucan chains. XTH belongs to the glycoside hydrolase family 16 (GH16) family (Yokoyama and Nishitani, 2001; Van Sandt et al., 2007; Eklöf and Brumer, 2010). XTHs regulate an array of physiological processes including the formation of secondary vascular tissue and the elongation of plant tissue (Miedes and Lorences, 2009; Harada et al., 2011).

Recently, XTHs have been shown to regulate the plant response to exogenous stress (Cho et al., 2006; Yang et al., 2011). For instance, AtXTH31 in roots is down-regulated by aluminum overexposure (Yang et al., 2011), and the overexpression of CaXTH3 improves salt and drought tolerance in transgenic *Arabidopsis* (Cho et al., 2006).

Along with the increased availability of the plant genome, the XTH family has been annotated in many plants and extensively researched (Yokoyama and Nishitani, 2001; Behar et al., 2018). In tomato, 56 XTH genes have been identified (Wang et al., 2018), 29 have been identified in rice, and 41 have been annotated in *Populus* (Yokoyama et al., 2004; Geisler-Lee et al., 2006; Saladié et al., 2006). A comprehensive cross-genome survey and phylogeny of the GH16 members revealed the evolutionary origin of EG16 and XTH proteins in a series of plant lineages including two citrus species (Behar et al., 2018). In the study, XTHs were extracted from genomic data (Wu et al., 2014) of *Citrus clementina* and *Citrus sinensis* based on Basic Local Alignment Search Tool (BLAST) strategy, which does not represent a specialized annotation process. The genomic data of *C. sinensis* used in studies from Behar and colleagues are yet to be assembled at the chromosome level, which brings uncertainty to subsequent research. In this study, we exhaustively annotated the CsXTHs from chromosome-level genomic data of *C. sinensis* (Xu et al., 2013) and performed an in-depth functional analysis.

Citrus bacterial canker (CBC) is caused by *Xanthomonas citri* subsp. *citri* (Xcc) (Schaad et al., 2005), which causes severe yield losses in citrus-producing regions globally. The cell wall is the initial barrier of the plant defense against bacterial infection and other stresses. In our previous long-term studies regarding the citrus transcriptomes induced by Xcc, XTH genes were identified as differentially expressed genes (DEGs) (**Supplementary Table 1**). In this study, we performed an *in silico* comprehensive detailed annotation and functional analysis of XTH genes in response to CBC by quantitative reverse transcriptase–polymerase chain reaction (qRT–PCR) and reverse genetics.

## Materials and Methods

### Comprehensive Annotation of XTHs in the Genome of Sweet Orange

For data mining, the proteome and genome of sweet orange were downloaded from *C. sinensis* annotation project (CAP) database (Xu et al., 2013; Wang et al., 2014) and Phytozome (Goodstein et al., 2012; Wu et al., 2014). A total of 33 XTH sequences of *Arabidopsis thaliana* were also collected from the Phytozome (Yokoyama and Nishitani, 2001; Goodstein et al., 2012). Exhaustive data mining and annotations were performed through a detailed three-step semi-automatic process that avoids the errors of automatic predictions (Fawal et al., 2014; Li et al., 2015). During the annotation process, Fgenesh++ (Solovyev et al., 2006), Pfam (El-Gebali et al., 2018) and Simple Modular Architecture Research Tool (SMART) (Letunic and Bork, 2018) were used for functional and structural re-annotation, while Scipio (Keller et al., 2008) was used to retrieve mis-annotated XTH genes in the automatic process and its corresponding chromosomal positions, gene structures, and sequences for all XTHs annotated. The records of XTHs in sweet orange were initially named “CsXTH” and followed by a number indicating its chromosomal order. EG16 (endoglucanases), a member of *C. sinensis* (CsEG16), was also annotated and analyzed with CsXTHs due to its relevance to CsXTHs (Behar et al., 2018). The EG16 member of *C. clementina* (Behar et al., 2018) was used as the query to survey the CsEG16s.

### Bioinformatics of the CsXTH Family

Maximum likelihood (ML) phylogeny was analyzed using Mega 7 (bootstrap = 500) with complete protein sequences aligned by Muscle (Kumar et al., 2016) and manually edited by BioEdit 2.0 (Tippmann, 2004). The phylogenetic tree was rooted by the BsMLGase of *Bacillus subtilis* (GenBank accession: WP_024571825.1, EC 3.2.1.73) (Michel et al., 2001). The exon-intronic structures of CsXTH genes were visualized with GSDS 2.0 (Gene Structure Display Server) (Hu et al., 2015), while chromosomal loci of the CsXTH genes were visualized with Mapchart 2.0 (Voorrips, 2002).

### Plant, Bacterial Materials, and Treatments

All plant materials were sampled from the National Citrus Germplasm Repository, Chongqing, China. Varieties Wanjincheng (*C. sinensis*) and calamondin (*Citrus madurensis*) were used for CBC and exogenous hormone assays. Wanjincheng was used for gene transformation. All plants were planted in a greenhouse at 28°C. An orange cultivation site in the Yunnan province, China, was the source of a variant of Xcc termed XccYN1 derived from sweet orange leaves that displayed a natural infection. Peptone-yeast extract-malt extract (PYM) with d-glucose 1.5% (w/v) and antibiotics at 28°C was used for the culture of Xcc bacteria.

### CBC and Exogenous Hormone Assay

For the analysis of the expression patterns of the CsXTHs, uniform and healthy fresh leaves excised from the plants were placed in culture plates containing sterile deionized water and maintained at 28°C with a 16-h light/8-h dark photoperiod. A 1,000-fold dilution of Xcc (OD600 = 0.5) was used to inoculate Wanjincheng and calamondin leaves in a 16-h light/8-h dark incubator at 28°C. Samples were collected at 0, 12, 24, 36, and 48 h postinoculation (hpi). To perform exogenous hormone assays, leaf discs were soaked in 10 μmol/L of salicylic acid (SA) or 100 μmol/L of methyl jasmonate (MeJA) and collected at 0, 6, 12, 24, 36, and 48 h posttreatment (hpt). Collected samples were assessed by qRT–PCR.

### Subcellular Localization Analysis

Subcellular localizations were analyzed with software prediction and transient expression. SignalP 4.0 (Petersen et al., 2011) and CELLO (Yu et al., 2006) were used for signal peptide and subcellular localization predictions, respectively. The coding sequence (CDS) of CsXTH04 lacking a stop codon was amplified with primers F-SC (GGGGTACCATGGCTTCTTTTCTATGGACTCT) and R-SC (TCCCCCGGGAATGTCACGGTCTCGTTTGCA) and inserted into the pLGNe-GFP vector to construct the transient expression vector. *Agrobacterium tumefaciens* EHA105 was used to infect onion epidermal cells, and green fluorescent protein (GFP) fluorescence was assessed after 48 h.

### Overexpression and Gene Silencing of CsXTH04

For overexpression plasmid construction, full-length CDS of CsXTH04 was amplified with primers (restriction sites included) F-OEc (CGGGATCCATGGCTTCTTTTCTATGGACT) and R-OEc (CGGAATTCTTAAATGTCACGGTCTCGTTTGCA) and inserted into pGLNe. For silencing vector construction, a 216-bp fragment was amplified with primers (restriction sites included) F-RIc (GCTCTAGAGGCGCGCCTTCATAGCCTCCTACAAGGGGT) and R-RIc (CGGGATCCATTTAAATGCATTCTGGTGAAGGCGTAGGGA) and integrated into the PUC-RANi vector. RNAi sequence including forward, intron, and reverse sequences were isolated and inserted into pLGNe to produce the final vector.

### Citrus Transformation and the Transgenic Characterization of Plants

Overexpression and silencing plasmids were introduced into *A. tumefaciens* EHA105 by heat shock. The shoot segments of Wanjincheng (*C. sinensis*) were transformed with *A. tumefaciens* using the Peng method (Peng et al., 2017). PCR and GUS (beta-glucuronidase) assays were used to confirm the presence of transgenic genes. Primers F-OEd (CGACACGCTTGTCTACTCCA) and R-OEd (TTAAATGTCACGGTCTCGTTTGCA) were used for overexpression plants; and F-RId (CTTCACCAGAATGCATTTAAATGTGTAA) and R-RId (GGTCTTACGGGATCCAAATACCTGCAAA) were used for silencing plants. The GUS activity in transgenic plants was detected using histochemical procedures (Sendín et al., 2017). Briefly, the leaf discs of transgenic citrus were infiltrated in X-GLUC solution (5-bromo-4-chloro-3-indolyl-beta-d-glucoronide) and incubated overnight at 37°C. Leaf discs were cleared by soaking in 70% ethanol and imaged by microscopy. After genomic PCR and GUS assays, qRT–PCRs were performed to analyze CsXTH04 expression in the positive plants. Wild-type (WT) plants were taken as controls for PCR verification, GUS assays, and qRT–PCR analysis.

### Assessment of Transgenic Plant Resistance Against Xcc

*In vitro* assays were performed to detect the Xcc resistance of transgenic plants as previously described (Peng et al., 2017). Six fully mature healthy leaves were used per transgenic plant. Briefly, six punctures were made in each of the leaves using a 0.5-mm-diameter pin, after which 1 µL of XccYN1 bacterial suspension (1 × 10^5^ CFU/mL) was inoculated at the sites. Images of the diseased areas were taken at 10 days postinoculation (dpi) and measured on ImageJ 2.0 software (National Institutes of Health, Bethesda, MD). The lesion sizes (LSs) of diseased spots and the disease index (DI) were used to evaluate Xcc resistance. The DI was calculated as previously described (Peng et al., 2017).

### RNA Isolation, cDNA Synthesis, and qRT–PCR Assay

Frozen tissues were ground in liquid nitrogen, and total RNA was extracted from leaf samples using miniprep kits purchased from AidLab Ltd (Beijing, China) based on the manufacturer’s protocols. RNA was reverse transcribed with TaKaRa kits (Dalian, China). The QuantStudio 7 (Applied Biosystems, USA) system and SYBR Premix ExTaq Green PCR kit (Bio-Rad, USA) were used for qRT–PCR with the actin gene of citrus (GenBank accession: GU911361.1) used as the internal control. Primer sequences were F-actin (GTTGCAGCAATGCCAGTGAA) and R-actin (GCGGCAGATGTGTTTTGTGT). The qRT–PCR parameters were as follows: 95°C for 5 min, followed by 40 cycles at 95°C for 10 s, 56°C for annealing, and extension for 30 s. A reaction mixture of 20 μL was composed of 100-ng cDNA, 0.5 μM of primers, and 10 μL of SYBR Green PCR mix. The 2^−∆∆CT^ method was used to evaluate the relative expression levels of each examined gene (Livak and Schmittgen, 2001). The primers for qRT–PCR were designed with Primer BLAST (**Supplementary Table 2**). For each gene, three biological and technical replicates were used.

### Statistical Analysis

SPSS V22 (SPSS, Inc., Chicago, USA) was used for all analyses. Gene expression differences were compared through an analysis of variance (ANOVA) *via* Fisher’s least significant difference (LSD) tests. Differences were considered statistically significant when *P < 0.05 and **P < 0.01. Each value represents the mean ± SD.

## Results

### Identification and Bioinformatics Analysis of CsXTHs

With exhaustive mining and detailed annotation, 34 XTH members were annotated and characterized, including 33 complete genes and a single pseudogene (CsXTH14) (**Table 1**). Among the XTH family, all genes were predicted by CAP, while 31 genes were predicted using the Phytozome protocol. Twelve XTH genes that were incorrectly predicted were manually annotated with Fgenesh++ re-annotation (Solovyev et al., 2006) and EST (expressed sequence tag) supports (**Table 1** and **Supplementary Table 3**). The members were named CsXTH01 to CsXTH34 based on the chromosomal order. To validate the annotation of CsXTHs, the EST database was downloaded from National Center for Biotechnology Information (NCBI) using the best EST hits. Finally, 17 genes were found with a total of 103 ESTs (**Table 1** and **Supplementary Table 3**). The ESTs helped the annotation of incorrectly predicted genes and predicted gene functions. Information on the CsXTH family, including gene name, ID, chromosome location, molecular weight (MW), amino acid length, isoelectric point (PI), and annotation modes are detailed in **Table 1**.

**Table 1:** Summary of XTHs and EG16 identified in the genome of sweet orange. All XTHs and EG16s are listed.

Name	CAP ID	Chr. Loci	EST No.	Intron No.	AA No.	MW (Dalton)	PI	Annotation
CsXTH01	Cs4g03180	Cs01:24073183.24074476 +	10	3	289	33,179.67	6.31	CAP, P, EST
CsXTH02	Cs1g21130	Cs02:4551552.4552810 −	5	3	293	34,116.17	5.02	CAP, P, M, EST
CsXTH03	Cs2g07590	Cs02:11822401.11823543 +	10	3	292	34,063.58	8.64	CAP, P, EST
CsXTH04	Cs2g14920	Cs02:14683444.14685405 +	11	3	334	38,119.86	6.56	CAP, P, EST
CsXTH05	Cs2g17920	Cs02:19614100.19616010 +	21	3	291	32,948.97	5.69	CAP, P, EST
CsXTH06	Cs2g22200	Cs03:12689865.12692080 +	1	3	356	40,363.55	8.86	CAP, P, EST
CsXTH07	Cs4g03050	Cs04:1564646.1565870 −	3	2	285	31,845.51	5.85	CAP, P, EST
CsXTH08	Cs4g03060	Cs04:1567748.1568823 −	3	2	286	32,012.71	6.21	CAP, P, EST
CsXTH09	Cs4g03080	Cs04:1582225.1583396 −	0	2	302	34,208.81	6.32	CAP, P
CsXTH10	Cs4g03110	Cs04:1592546.1593612 +	0	2	266	30,142.09	8.61	CAP, M
CsXTH11	Cs4g03120	Cs04:1595185.1596639 +	0	3	280	32,068.14	9.17	CAP, P, M
CsXTH12	Cs4g03130	Cs04:1599619.1600737 −	1	2	285	31,743.37	5.85	CAP, P, EST
CsXTH13	Cs4g03140	Cs04:1602516.1603589 −	1	2	286	32,074.8	6.21	CAP, P, EST
CsXTH14*	Cs4g03145	Cs04:1603378.1605201 +	0	2	122	N/A	N/A	CAP, M
CsXTH15	Cs4g03150	Cs04:1607036.1608207 −	0	2	302	34,218.8	6.32	CAP, P
CsXTH16	Cs3g08950	Cs04:1620024.1621096 +	0	2	291	33,183.25	8.64	CAP, P, M
CsXTH17	Cs4g03190	Cs04:1622154.1623610 +	0	2	267	30,662.64	8.27	CAP, P
CsXTH18	Cs4g03200	Cs04:1626153.1627263 +	10	2	283	32,147.03	7.6	CAP, P, EST
CsXTH19	Cs4g03210	Cs04:1630852.1631877 +	1	2	280	31,656.59	8.77	CAP, P, EST
CsXTH20	Cs4g03220	Cs04:1635451.1637865 +	2	3	265	30,083.89	9.18	CAP, P, M, EST
CsXTH21	Cs4g03230	Cs04:1655583.1656783 +	0	2	295	33,647.46	4.91	CAP, P
CsXTH22	Cs4g03240	Cs04:1659586.1661426 +	0	3	288	33,375.61	8.62	CAP, P, M
CsXTH23	Cs4g16330	Cs04:15956333.15957867 −	0	3	291	33,295.65	9.01	CAP, P
CsXTH24	Cs5g27840	Cs05:30304069.30306657 −	0	3	288	33,413.58	7.71	CAP, P, M
CsXTH25	Cs6g02160	Cs06:1715203.1716021 −	0	0	272	31,261.97	4.75	CAP
CsXTH26	Cs6g16990	Cs06:17595562.17596413 −	0	0	283	31,807.91	7.08	CAP, P
CsXTH27	Cs7g08460	Cs07:5311346.5312496 −	3	2	314	34,804.51	8.47	CAP, P, EST
CsXTH28	Cs8g03550	Cs08:1713946.1715132 −	0	3	304	35,052.57	8.78	CAP, P
CsXTH29	Cs8g12020	Cs08:13532542.13534098 +	3	2	293	34,239.08	9.03	CAP, P, M, EST
CsXTH30	Cs8g15720	Cs08:18866226.18867848 −	2	3	296	33,394.4	6.49	CAP, P, EST
CsXTH31	orange1.1t00547	CsUn:6505748.6506605 −	0	0	285	32,501.44	5.65	CAP, P
CsXTH32	orange1.1t00876	CsUn:12458388.12460216 +	0	3	293	33,986.6	9.13	CAP, P, M
CsXTH33	orange1.1t02385	CsUn:35972640.35973816 +	16	2	288	33,082.38	9.11	CAP, P, M, EST
CsXTH34	orange1.1t02575	CsUn:39382705.39383552 +	0	3	282	33,157.23	7.76	CAP, P, M
CsEG16	orange1.1t00297	CsUn:4793451.4794742 +	0	1	215	24,208.07	4.92	CAP, P

AA, amino acid; MW, molecular weight; PI, isoelectric point; *Pseudogene. +/−, Directions of each gene on chromosomes; P, Prediction by Phytozome; CAP, Prediction by Citrus sinensis annotation project; M, Manual correction; EST, Genes with EST hits; XTH, xyloglucan endotransglucosylase/hydrolase.

**Figure 1:**
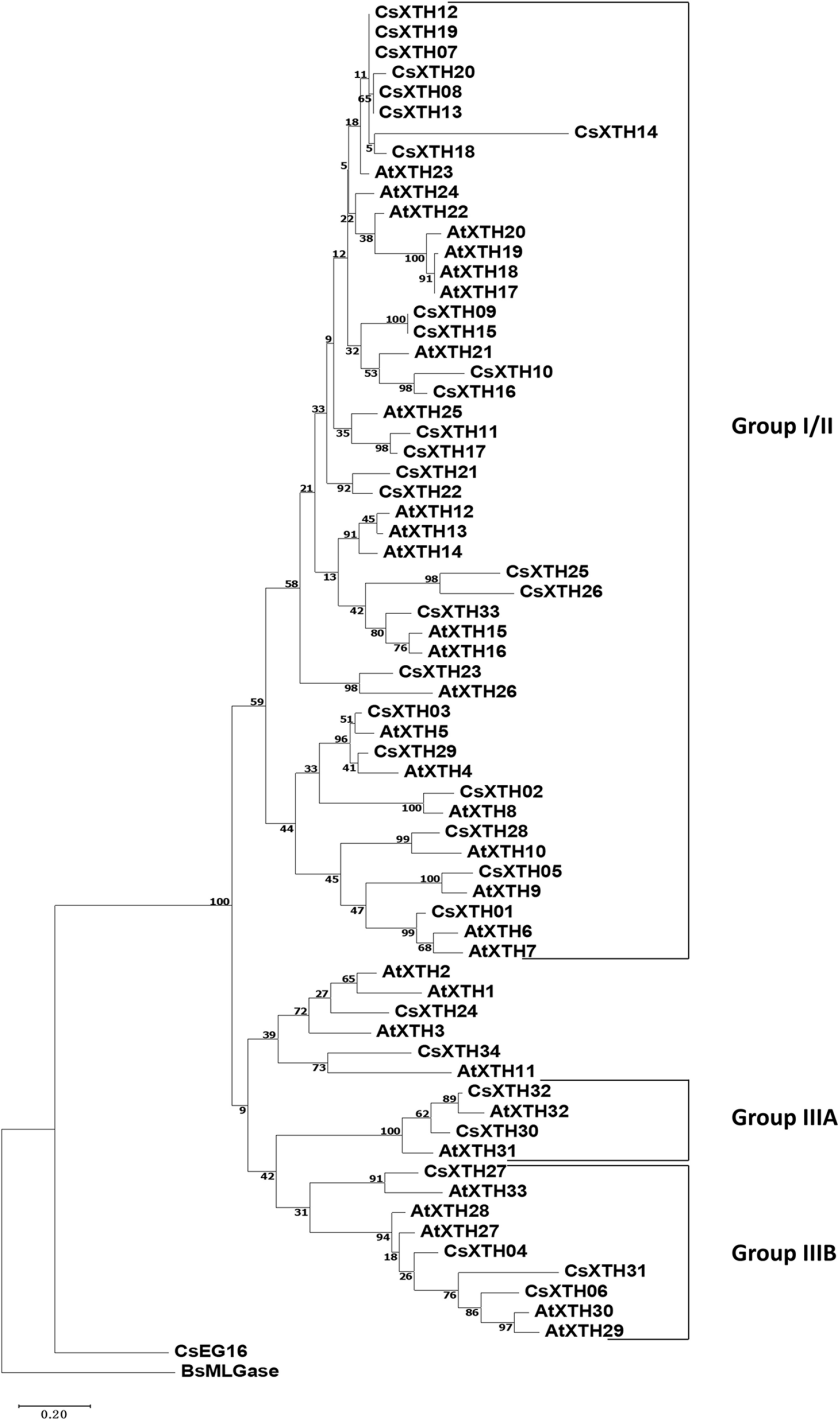
Rooted maximum-likelihood (ML) phylogeny of XTHs and EG16 from sweet orange and *Arabidopsis thaliana*. All complete XTHs and EG16s from *A. thaliana* and sweet orange were used for ML tree construction using MEGA 7 with bootstrap = 500. Sub-family assignments are indicated on the right. Trees were rooted by BsMLGase. XTH, xyloglucan endotransglucosylase/hydrolase.

The complete amino acid sequences of CsXTHs (**Supplementary Table 4**) and AtXTHs (**Supplementary Table 5**, **Supplementary Files 1 and 2**) were used for phylogenetic analysis. The rooted ML phylogenetic tree indicated that the CsXTHs can be divided into I/II, IIIA, and IIIB groups (**Figure 1**) according to the clade identifiers used in AtXTHs (Yokoyama et al., 2010).

Exon-intronic analysis was performed for the CsXTH genes, which revealed no introns in three genes (CsXTH25, CsXTH26, and CsXTH31). In other genes, the intron numbers ranged from 2 (15 genes) to 3 (15 genes). The introns and exons were well conserved between closely related genes. For example, closely related CsXTH, CsXTH25, and CsXTH26 genes contained no introns (**Supplementary Figure 1A**). With respect to functional annotations, all CsXTHs excluding CsXTH25 and CsXTH26 possessed an N-terminal glyco hydro 16 domain (Pfam: PF00722) and a C-terminal XET-C domain (Pfam: PF06955) (**Supplementary Figure 1B**).

### Chromosomal Distribution and Duplication Refer to a Duplication Hot Spot on Chromosome 4

**Figure 2:**
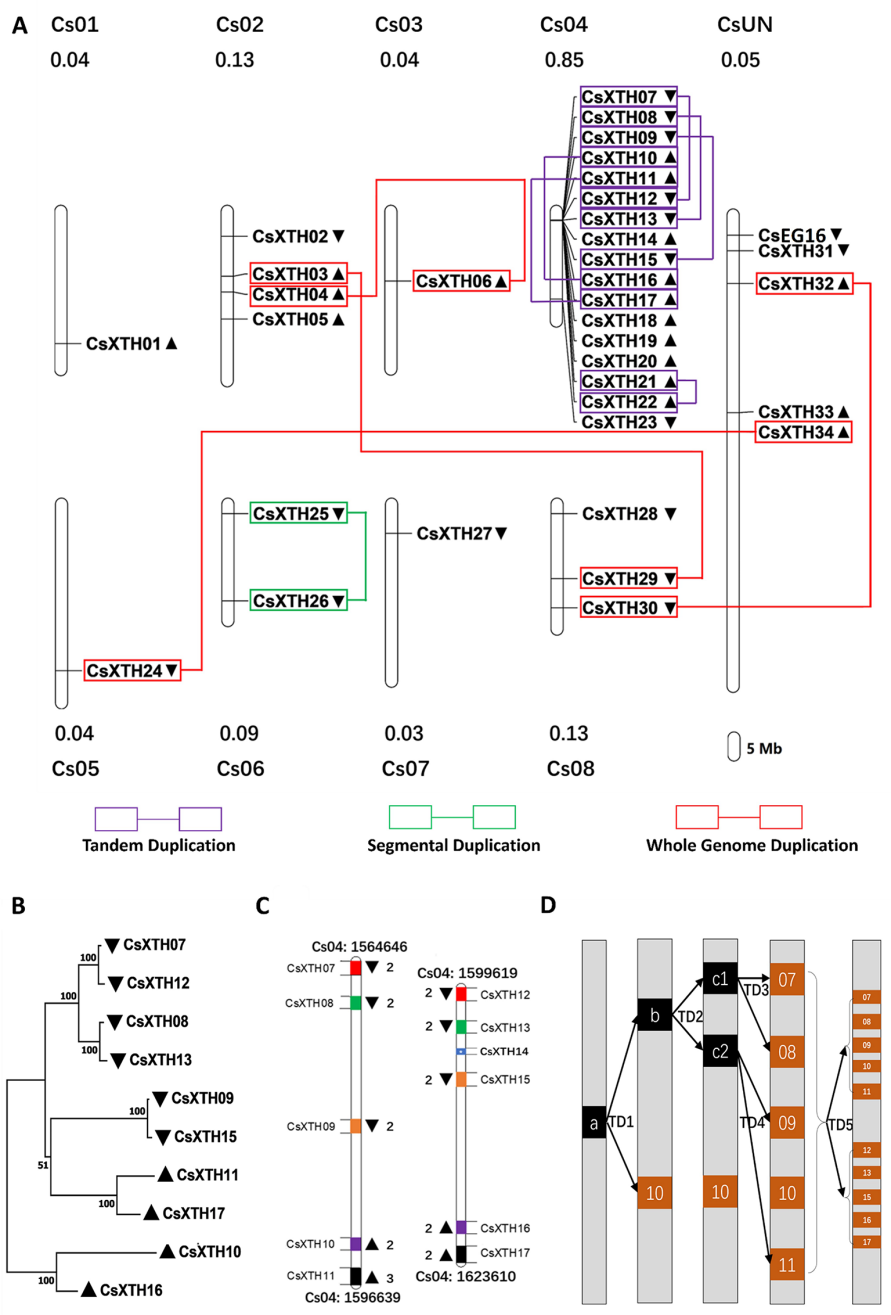
Chromosomal loci and duplications hot spots of CsXTH genes. **(A)** Chromosomal locations and duplications of CsXTH and CsEG16 genes. The map was visualized by MapChart 2.1, and the concentration of CsXTH genes (number of CsXTHs per Mb) are written above each chart. Gene orientations are shown in nearby triangles. **(B)** Maximum-likelihood phylogeny of duplicated CsXTH genes on chromosome 4 (MEGA 7, bootstrap = 500). **(C)** Comparison of two duplicated segments. Gene sizes and intervals are to scale. Start and stop positions of the duplicated segments were written on the top and bottom of the segments. Gene orientations are shown with triangles on the left and right. Homologous genes are shown in the same color on the two duplicated segments. **(D)** Model for the evolutionary history of the two duplicated segments. a, b, and c represent ancestral genes during the evolution process. 07–17 represent CsXTH07–CsXTH17, respectively.

The CsXTH genes were distributed among all chromosomes excluding chromosome 9 based on their positions in the genome of sweet orange (**Figure 2A**). Chromosome 4 contained the highest number of CsXTHs (17 members). By contrast, only a single CsXTH on chromosomes 1, 3, 5, and 7 was identified. Chromosome 4 showed the highest density of CsXTH genes (0.85 per Mb), while chromosomes 1 and 3 showed the lowest density. In addition, the distribution of CsXTH genes was uneven. A higher density of CsXTH was found in specific chromosomal regions, mainly on chromosome 4 (**Figure 2A**). To further understand how CsXTH genes evolved, gene duplication events were investigated in sweet orange. In this study, 10 pairs of duplications were arranged in blocks of duplicates, among which were a single pair of segmental duplications (SDs), six pairs of tandem duplications (TDs), and three pairs of whole-genome duplications (WGDs) (**Figure 2A**). These results strongly indicated that TD and WGD make major contributions to the expansion of the XTH family in sweet orange. According to the phylogenetic tree of CsXTHs (**Figure 1**) and the identified duplications, a large region on chromosome 4 was detected. In the 58,964-bp region (chromosome 04: 1564646 to 1623610), 11 CsXTHs were involved in the segment (CsXTH07 to CsXTH17) (**Figure 2A**). A phylogeny (ML) with 10 CsXTHs (pseudogene CsXTH14 was not used for phylogeny) showed five pairs of duplicated genes (CsXTH07–CsXTH12, CsXTH08–CsXTH13, CsXTH09–CsXTH15, CsXTH10–CsXTH16, and CsXTH11–CsXTH17) (**Figure 2B**). The duplicated genes had the same directions on chromosomes, similar gene sizes, and the same number of introns except for CsXTH11–CsXTH17 (**Figure 2B**). The duplicated genes in this region produced two duplicated segments (**Figure 2C**). There was one unique case with an interval between CsXTH08 and CsXTH09 that differed from the interval between CsXTH13 and CsXTH15. We reasoned that these differences were due to the pseudogenization process of CsXTH14. In conclusion, a hypothesis for the evolution of the 10 CsXTH genes was constructed. One ancestral gene duplicated four times and formed the CsXTH07-to-CsXTH11 segment. A segment of CsXTH07 to CsXTH11 underwent TD and was inserted into a nearby loci on chromosome 4 to form the segment CsXTH12 to CsXTH17 (**Figure 2D**).

### CsXTHs Are Involved in the Response to Xcc Infection

**Figure 3:**
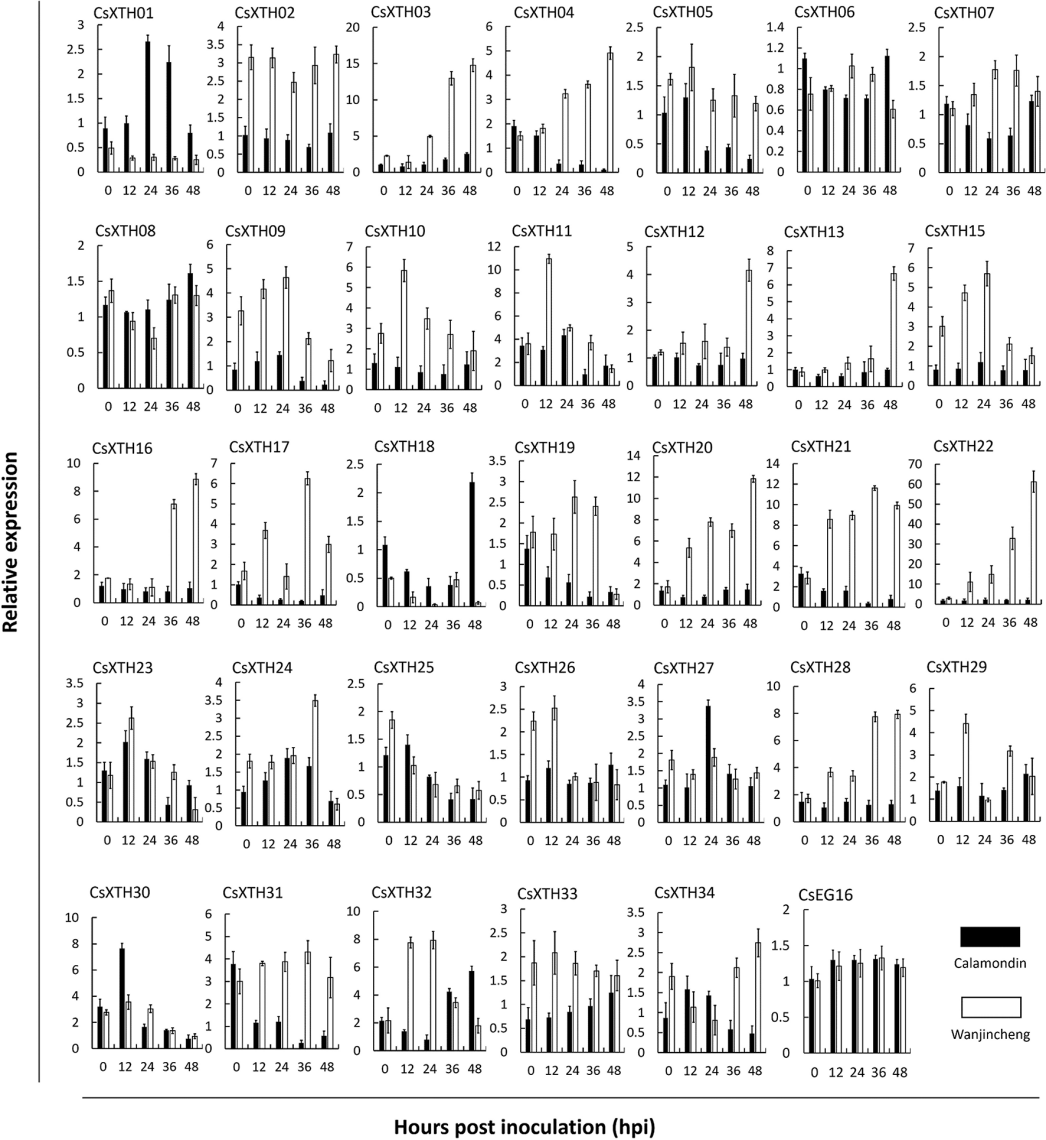
Differential expression profiles of CsXTHs induced by Xcc. Samples for expression profiling were collected from Wanjincheng (empty bars) and calamondin (solid bars) at 0, 12, 24, 36, and 48 h post inoculation (hpi) of Xcc. Expression profiles were detected by qRT–PCR and normalized to CsActin. Control samples were inoculated with LB medium. Note that the relative expression of CsXTHs was on a different scale. LB, lysogeny broth; qRT–PCR, quantitative reverse transcriptase–polymerase chain reaction; Xcc, *Xanthomonas citri* subsp. *citri*.

To further explore CsXTH functions, the expression of these XTHs together with CsEG16 during the induction of biotic stress was investigated. To detect the involvement of CsXTHs in Xcc infection, their expression at 0, 12, 24, 36, and 48 hpi was detected by qRT–PCR. From the qRT–PCR data, different expression profiles were detected for each gene (**Figure 3**). During Xcc infection, a group of CsXTHs with up-regulated expression profiles in the citrus canker sensitive variety Wanjincheng were identified including CsXTH03, CsXTH04, CsXTH12, CsXTH13, CsXTH16, CsXTH20, CsXTH21, CsXTH22, and CsXTH28. Among these genes, CsXTH04 and CsXTH21 showed a down-regulated profile in the citrus canker-resistant variety calamondin. This gene, therefore, represents an Xcc susceptibility gene. Based on our data mining and annotation together with Xcc-induced expression analyses, we thus identified CsXTH04 as a candidate gene for further CBC studies.

### CsXTH04 Is an Extracellular Protein

**Figure 4:**
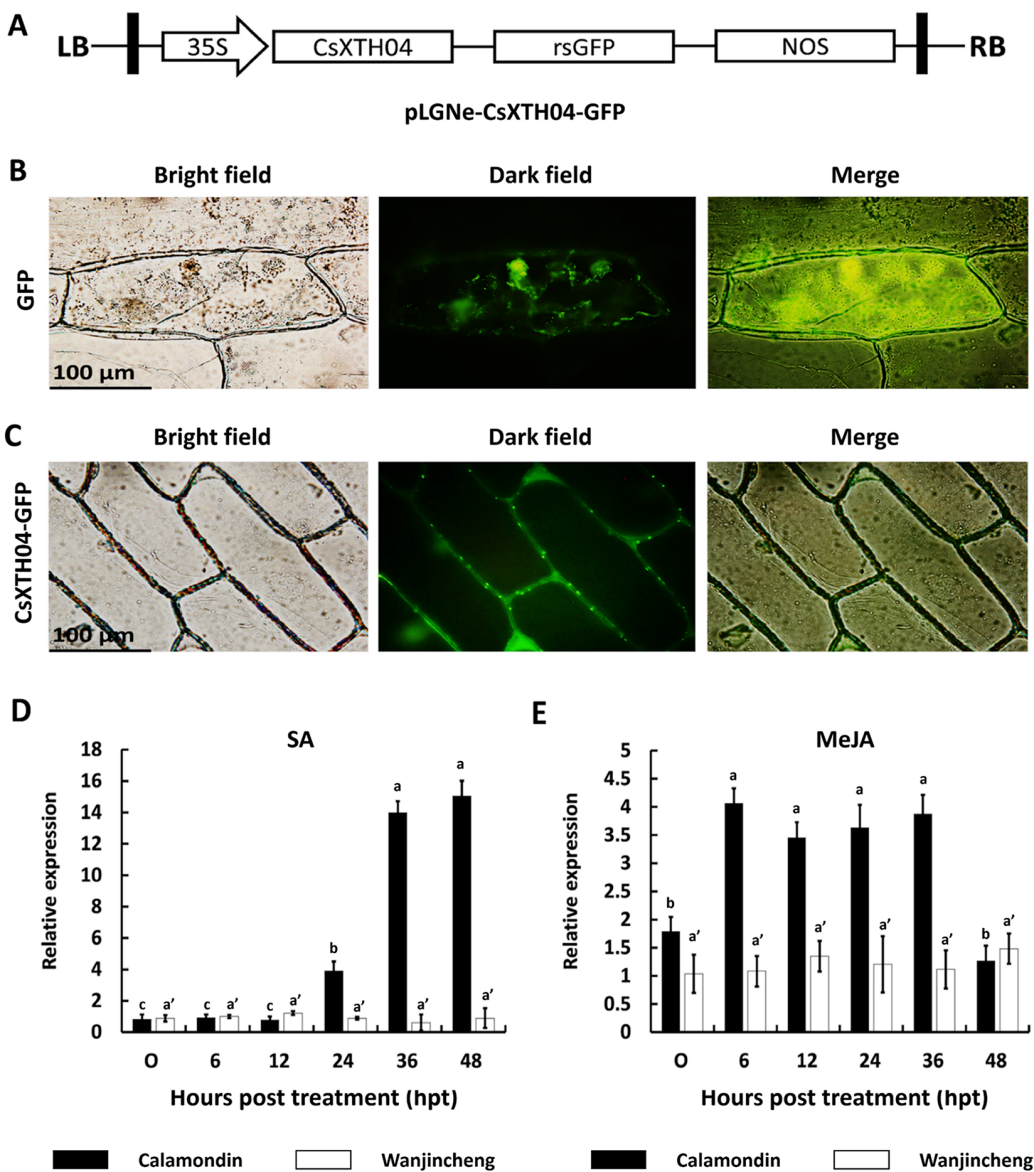
Subcellular localization and SA/MeJA inducible expression profiles of CsXTH04. **(A)** Structure of the transient expression plasmid (pLGNe-CsXTH04-GFP). LB, left border; RB, right border; 35S, CaMV35S promoter; NOS: terminator. **(B)** Transient expression of GFP. **(C)** Transient expression of CsXTH04-GFP. Each field of view is displayed as a bright field, dark field, and merged image. Expression of CsXTH04 induced by SA **(D)** and MeJA **(E)** was measured by qRT–PCR and normalized to CsActin. Samples were collected at 0, 6, 12, 24, 36, and 48 hpt with SA and MeJA. In **(B)** and **(C)**, scale bars = 100 μm. In **(D)** and **(E)**, solid bars represent calamondin, while empty bars represent Wanjincheng. Results were analyzed *via* Tukey’s honestly significant differences (*P* = 0.05) in three biological replicates. Each value represents the mean ± SD. For **(D)** a = 0.213, b = 1.000, c = 0.979, a' = 0.597. **(C)** a = 0.702, b = 0.380, a' = 0.845. GFP, green fluorescent protein; MeJA, methyl jasmonate; qRT–PCR, quantitative reverse transcriptase–polymerase chain reaction; SA, salicylic acid.

CsXTH04 was identified by qRT–PCR as a candidate gene involved in Xcc infection (**Figure 3**). We, therefore, investigated the function of CsXTH04. The subcellular localization of CsXTH04 was determined by software predictions and exogenous transient expression systems. The protein contained a 23-aa signal peptide (MVVSYEGCFLLVFSLLAVVASGL) at the N-terminal, suggesting it to be secreted from cells. Potential extracellular loci were also identified (**Supplementary Table 6**). To validate these predictions, we examined the subcellular localization of CsXTH04 following transient expression of a recombinant pLGNe-CsXTH04-GFP plasmid (**Figure 4A**). Both the cytoplasm and nucleus of the control exhibited green fluorescence (**Figure 4B**), but the strongest GFP fluorescence was observed at the plasma membrane and cell wall of the epidermal onion cells with plasmid pLGNe-CsXTH04-GFP (**Figure 4C**). Both predictions and transient expression analysis, therefore, indicated that CsXTH04 performs extracellular functions.

### Csxth04 Expression Was Induced by SA and MeJA

Proteins associated with plant disease are often regulated by phytohormones (Zuo et al., 2015; He et al., 2019). To assess the involvement of CsXTH04 in disease resistance-related signaling pathways, SA and MeJA inductions were performed, and CsXTH04 expression was detected by qRT–PCR. The results showed that the expression of CsXTH04 in calamondin in response to exogenous SA increased ∼14-fold during treatment, while in Wanjincheng, the expression was unaffected within the 48-h treatment period (**Figure 4D**). Regarding MeJA induction, the expression of CsXTH04 also increased ∼3-fold in calamondin. After 36 hpt, the expression levels decreased to below starting levels. Within the 48-h MeJA treatment period, CsXTH04 showed no significant differences in Wanjincheng (**Figure 4E**). From these results, we concluded that in calamondin, CsXTH04 expression is be induced by both SA and MeJA, while in Wanjincheng, CsXTH04 expression could not be induced.

### Overexpression of CsXTH04 Confers CBC Susceptibility to Transgenic Plants

**Figure 5:**
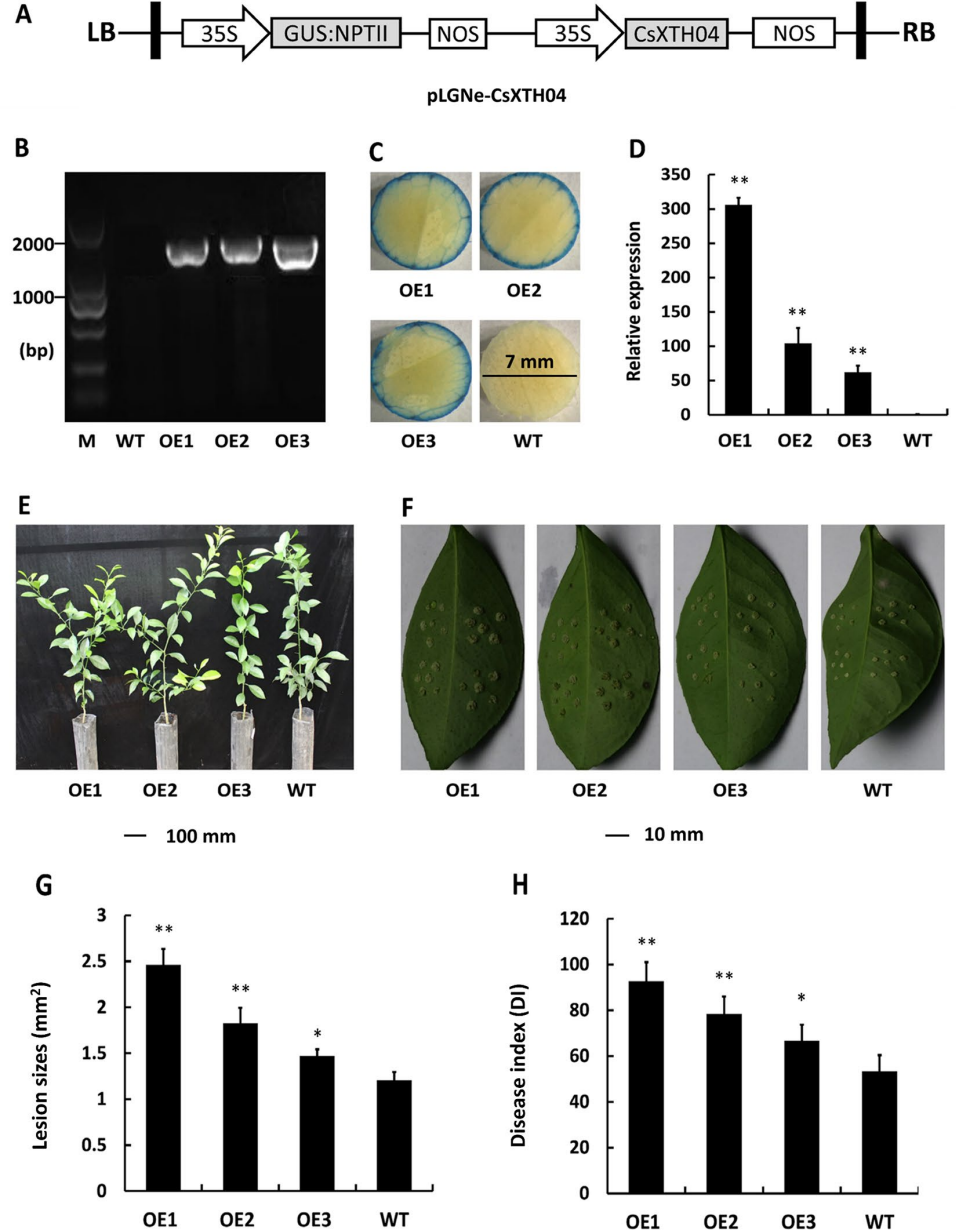
Characterization and evaluation of CsXTH04 overexpression plants in response to Xcc. **(A)** Structure of the plasmids for overexpression assays (pLGNe-CsXTH04). LB, left border; RB, right border; 35S, CaMV35S promoter; NOS: terminator. **(B)** Validation of transgenic plants by PCR. **(C)** Validation of transgenic plants by GUS assays (leaf disc diameter = 7 mm, staining period = 24 h). **(D)** Overexpression of CsXTH04 detected by qRT–PCR. **(E)** Phenotypes of transgenic plants. **(F)** Disease symptoms on leaves of overexpressed transgenic and WT plants inoculated by Xcc. Sampling and imaging at 10 dpi. Lesion sizes (LSs) **(G)** and disease index (DI) **(H)** of each transgenic plant were assessed to evaluate disease resistance. In **(D)**, **(G)**, and **(H)**, *P < 0.05, **P < 0.01 Student’s *t*-test. Each value represents the mean ± SD. In **(B)** to **(H)**, OE1–OE3 indicate overexpression plants; WT, wild type. In **(B)**, M indicates DNA marker. qRT–PCR, quantitative reverse transcriptase–polymerase chain reaction; Xcc, *Xanthomonas citri* subsp. *citri*.

Transgenic citrus plants were produced to aid our understanding of the role of CsXTH04. The CsXTH04 overexpression plasmid contained a GUS coding sequence and CaMV 35S promoter (**Figure 5A**). Three plants (OE1 to OE3) with the transgenic integration of CsXTH04 were confirmed by genomic PCR and GUS assays. Compared with WT, OE1 to OE3 had a 1.8-kb signature fragment that was detected by PCR (**Figure 5B**). The OE1 to OE3 showed a blue color on the edge of the leaf discs, while WT leaves showed no color (**Figure 5C**). Following qRT–PCR detection, the three overexpressed plants possessed high levels of CsXTH04 (300-, 100-, and 55-fold higher than WT, respectively) (**Figure 5D**). Regarding phenotypes, the transgenic plants exhibited normal growth rates than did WT plants (**Figure 5E**). To study the CBC resistance of the overexpressed transgenic plants OE1 to OE3, *in vitro* assays were performed through the inoculation with pinpricks. The sizes of the lesions on OE-transgenic leaves were larger than those of WT plants (**Figure 5F**). The pustules of infection by Xcc were exacerbated by the overexpression of CsXTH04, while the highest levels of susceptibility were displayed by OE1, followed by OE2 and OE3. Regarding the LS, OE1 possessed the largest lesions, which were ∼2.36-fold higher than those of WT, while OE3 possessed the lowest, which were 1.39-fold higher than those of WT (**Figure 5G**). DI, as a measure of disease severity, was enhanced by 36.7–87.8% in the transgenic lines compared with WT (**Figure 5H**). We, therefore, concluded that the overexpression of CsXTH04 conferred Xcc suppressibility to transgenic citrus.

### Silencing of CsXTH04 Confers CBC Resistance to Transgenic Plants

**Figure 6:**
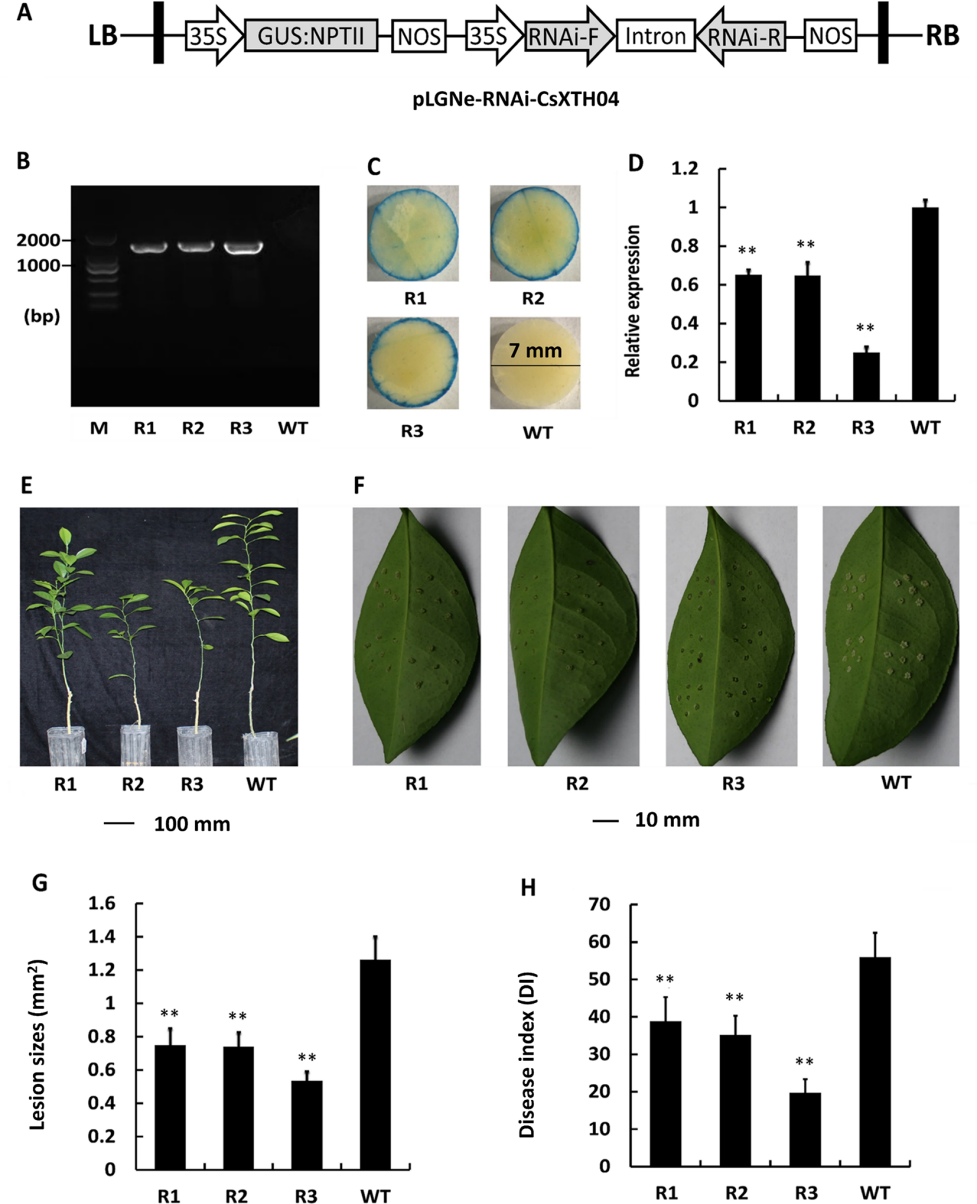
Characterization and evaluation of the silencing of transgenic plants in response to Xcc. **(A)** Plasmid structures for silencing assays by RNAi (pLGNe-RNAi-CsXTH04). LB, left border; RB, right border; 35S, CaMV35S promoter; NOS, terminator. **(B)** Validation of transgenic plants by PCR. **(C)** Validation of transgenic plants by GUS staining (leaf disc diameter = 7 mm, staining period = 24 h). **(D)** Expression of CsXTH04 detected by qRT–PCR. **(E)** Phenotypes of transgenic and WT plants. **(F)** Disease symptoms on the leaves of silenced transgenic plants and WT plants inoculated by Xcc. Sampling and imaging at 10 dpi. Lesion sizes (LSs) **(G)** and disease index (DI) **(H)** of each transgenic plant were assessed to evaluate disease resistance. In **(D)**, **(G)**, and **(H)**, **P < 0.01, Student’s *t*-test. Each value represents the mean ± SD. In **(B)** to **(H)**, R1–R3 indicate silencing plants; WT, wild type. In **(B)**, M indicates DNA marker. qRT–PCR, quantitative reverse transcriptase–polymerase chain reaction; Xcc, *Xanthomonas citri* subsp. *citri*.

To further elucidate the role of Wanjincheng, CsXTH04 was silenced using RNAi approaches. RNAi sequences, including forward, intron, and reverse sequences, were digested and inserted into pLGNe (**Figure 6A**). To verify the transgenic plants, genomic PCRs were performed, and three silenced plants were obtained (**Figure 6B**). The plants were subjected to verification by GUS stain (**Figure 6C**). The three plants (R1, R2, and R3) exhibited relatively low levels of expression of CsXTH04 than did WT plants (41%, 40%, and 23% of WT), which was validated by qRT–PCR (**Figure 6D**). Compared with WT, the three silenced plants exhibited normal growth states (**Figure 6E**). The use of inoculation by pinprick was applied to study the CBC resistance of the three silenced mutant plants. Transgenic plants R1 to R3 exhibited smaller-sized pustules than did WT (**Figure 6F**). We, therefore, conclude that silencing CsXTH04 significantly enhances its resistance to Xcc infection. Statistically diseased lesions in the three silenced plants (R1, R2, and R3) showed smaller dimensions when examined against WT (58%, 57%, and 42%, respectively) (**Figure 6G**). The analysis of CBC severity revealed that the three silenced mutant plants had significantly higher DI than WT (**Figure 6H**). Consequently, disease severity decreased by 29% (R1) to 65% (R3). As such, CsXTH04 mutant’s exhibit increased CBC resistance, suggesting that CsXTH04 is an Xcc susceptibility gene.

## Discussion

Xyloglucan is a hemicellulose polysaccharide that is a major constituent of the primary cell walls of dicots and some monocots (Vogel, 2008; Scheller and Ulvskov, 2010; Pauly and Keegstra, 2016). XTHs can cut and rejoin xyloglucan to regulate the organization and composition of the cell wall during plant development and stress responses, including abiotic and biotic stresses (Yokoyama and Nishitani, 2001). To date, numerous XTHs genes have been identified from plants (Behar et al., 2018) including 33 genes in *Arabidopsis* (Rose et al., 2002), 29 in rice (Yokoyama et al., 2004), 56 in tomato (Wang et al., 2018), and 44 in *Medicago* (Xuan et al., 2016). Despite the XTH family of two *Citrus* species (*C. clementina* and *C. sinensis*) being surveyed from genomic data, the XTHs have not been annotated in a detailed manner. The genes extracted from genomics using automated BLAST-based protocols typically contain errors and/or redundancy (Fawal et al., 2014; Li et al., 2015). In this study, 12/34 XTH genes were incorrectly predicted and manually corrected (**Table 1**). At the chromosome level, assembled genomic datasets (Xu et al., 2013) are in contrast to those described in previous studies (Behar et al., 2018).

The number of XTH genes in sweet orange was higher than that of *Arabidopsis* and rice but lower than that of tomato and *Medicago*, suggesting that this was a result of plant-specific gains and losses (Song et al., 2015). On chromosome 4 of sweet orange, there is a CsXTH hot spot with 10 duplicated genes. This duplication hot spot contains TDs combined with segmental duplications (**Figure 2A**). The up-regulation of CsXTH was subject to protein concentrations for specific functional requirements or functional differentiation (Li et al., 2015). This duplication hot spot of CsXTHs represents research interests on future functional and evolutionary studies.

Plant cell walls play an important role in the regulation of environmental stresses. Plant XTHs are important cell wall-modifying enzymes that participate in cell wall extension and degradation, maintaining the integrity and strength of the cell wall under normal and stressful environments (Zhu et al., 2012). Compared with abiotic stresses, the relationship between XTHs and plant disease, particularly CBCs, is poorly defined. In our experience of studying the transcriptomes of species infected with Xcc, XTHs were consistently represented (**Supplementary Table 1**), indicating the involvement of XTHs in Xcc infection. To investigate the XTH genes that potentially respond to CBC, we performed qRT–PCR to detect the induced expression of CsXTHs. We found that CsXTH04 was involved in Xcc infection and SA/MeJA signaling pathways, which preliminarily validated their links to disease. The differential induction of Wanjincheng and calamondin provide insight into the different cis-elements between these two species (Song et al., 2016). Our present work highlighted the involvement of three CsXTH genes in CBC resistance, further extending the list of CBC-related genes, confirming a role for XTHs in pathogen infection. CsXTH04 was investigated in depth using overexpression and silencing strategies. We found that the overexpression of CsXTH04 conferred CBC suppressibility to transgenic citrus, while the silencing of CsXTH04 conferred CBC resistance (**Figures 5** and **6**). However, many questions remain unsolved and require further investigation. These include the functional mechanisms of CsXTH04 in CBC infection and how bacteria induce the expression of CsXTH04. Further physiological and molecular/genetic studies of CsXTH04 must now be performed to clarify its role in the plant response to CBC stress and tolerance.

## Conclusions

To our knowledge, this is the first focused annotation and expression profiling of XTHs in citrus in response to Xcc induction. In the present study, 34 XTHs from the genome of sweet orange were annotated, providing an initial basis for the functional investigation and evolutionary study of this family. The expression profiling of CsXTH induced by Xcc provides insight into the involvement and development of CBC. The CBC identified genes, including CsXTH04, hold value for citrus breeding to improve CBC resistance.

## Data Availability

All the datasets generated for this study are included in the **Supplementary Files**.

## Author Contributions

QLi, SC and YH designed the experiments; QLi, AH, JQ, WD, YH, QLo, and XZ performed the experiments; QLi, TL and LY performed data analysis; SC and YH contributed materials and tools for analysis and reagents; QLi wrote this manuscript; All authors read and approved the final manuscript.

## Funding

This study was funded by the National key Research and Development Program of China (2018YFD1000306), Fundamental Research Funds for the Central Universities (SWU115025), Earmarked Funds for the China Agriculture Research System (CARS-26), Opening Project of the Key Laboratory of Horticulture Science for Southern Mountainous Regions, Guangxi Science and Technology Key Project (GuiKeAA18118046-6).

## Conflict of Interest Statement

The authors declare that the research was conducted in the absence of any commercial or financial relationships that could be construed as potential conflicts of interest.
