# Supplementary material for: Systematic Analysis and Functional Validation of Citrus XTH Genes Reveal the Role of Csxth04 in Citrus Bacterial Canker Resistance and Tolerance
Source: Front Plant Sci. 2019 Sep 27;10:1109. doi: 10.3389/fpls.2019.01109 (PMC6776591; doi:10.3389/fpls.2019.01109)

**Figure S1. Exon-intron structures and functional domains of CsXTHs.** (A) Exon-intron organization of CsXTHs visualized by GSDS 2.0. Exons and introns are indicated by blue boxes and dashed lines respectively. (B) Functional domains in CsXTHs analyzed by Pfam.


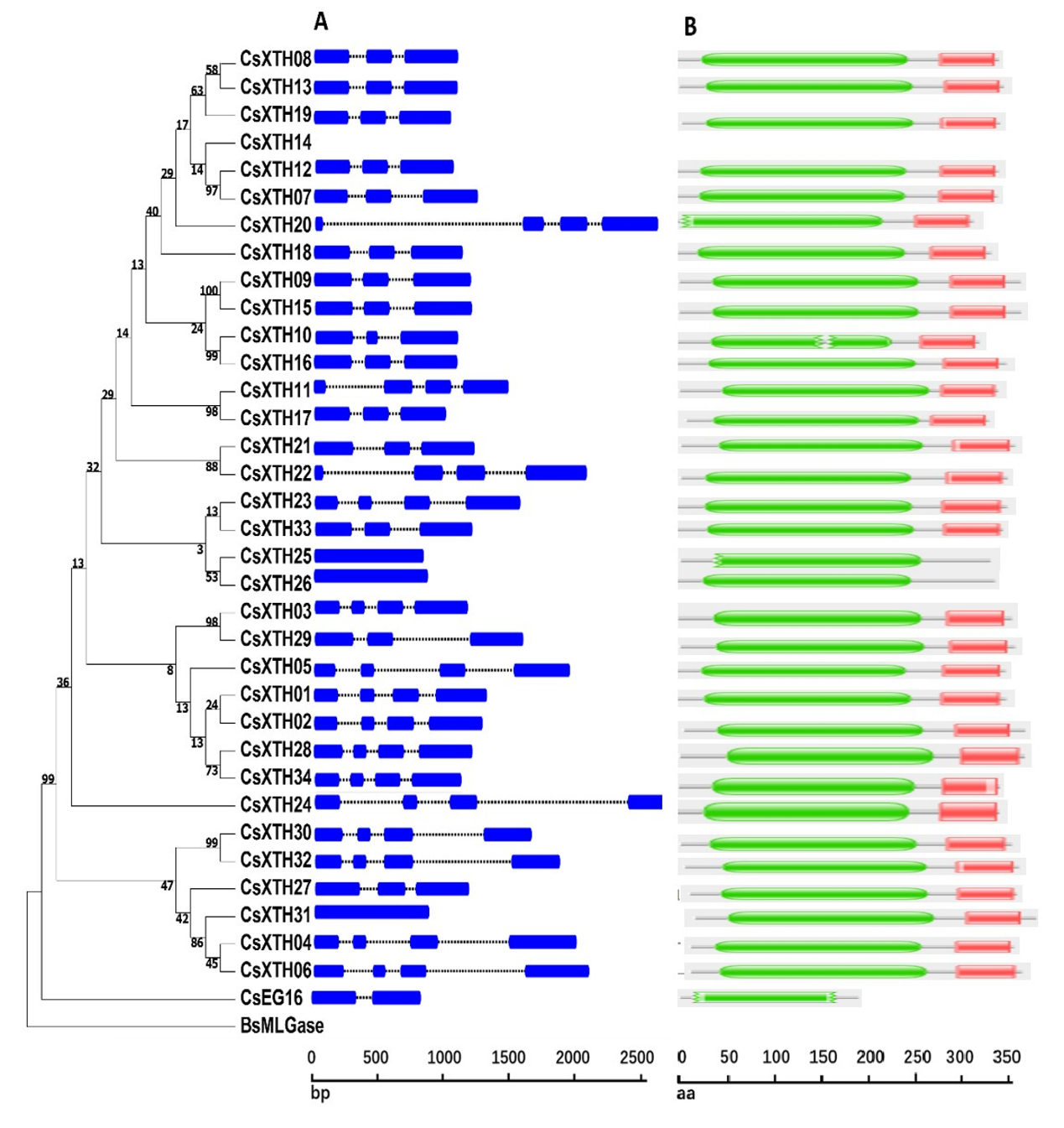

Supplement: Supplementary file 3 [file Table_2.docx]
